# Supplementary material for: Beneficial microbiome and diet interplay in early-onset colorectal cancer
Source: EMBO Mol Med. 2024 Dec 9;17(1):9–30. doi: 10.1038/s44321-024-00177-0 (PMC11730345; doi:10.1038/s44321-024-00177-0)
Supplement: Supplementary file 1 — Appendix [file 44321_2024_177_MOESM1_ESM.pdf]

Beneficial Microbiome and Diet Interplay in Early-Onset Colorectal Cancer

Zhengyuan Zhou[1,†], Linda Kleis[2,†], Ana Depetris-Chauvin[1,†], Stefanie Jaskulski[3], Victoria Damerell[4], Karin B. Michels[3], Biljana Gigic[4], Ute Nöthlings[2,*], Gianni Panagiotou[1,5,6,*]

Content

Appendix Table S1 - Studies investigating the association between diet and early-onset colorectal cancer and early-onset- precursors/ adenomas……………………………………………………………………………………1

Appendix Table S1 - Studies investigating the association between diet and early-onset colorectal cancer and early-onset- precursors/ adenomas.

Author (year), Country	Participants (n, age, sex)	Dietary assessment	Findings	Potential dietary risk factors
**Prospective cohort studies**				
Kim et al.<br>(2023)<br>USA	• EO-CRC cases: n = 349<br>• follow-up: 2 202 604 person-years<br>• 25-42 years at enrolment and < 55 years at diagnosis<br>• females	• validated, semi-quantitative FFQ every 4 years	• inverse association between total calcium intake and EO-CRC (HR per 300 mg/day increase 0.87; 95% CI 0.75–1.00)	• low calcium intake
Hur et al.<br>(2021)<br>USA	• EO-CRC cases: n = 109<br>• follow-up: 1 358 142 person-years<br>• 25-42 years at enrolment and < 50 years at diagnosis<br>• females	• validated, semi-quantitative FFQ every 4 years<br>• high school FFQ (adolescent diet (1960 – 1982))	• consumption of ≥2 SSB/day in adulthood -> doubled risk of EO-CRC (RR 2.18; 95% CI 1.10 - 4.35) compared to < 1 SSB/week<br>• per serving/day increase: 16% higher risk (RR 1.16; 95% CI 1.00 - 1.36)<br>• per serving/day increase of SSB age 13–18 years: 32% higher risk (RR 1.32; 95% CI 1.00 - 1.75)	• higher intake of SSB
Joh et al.<br>(2021)<br>USA	• EO-adenoma cases: n = 2909<br>• 25-42 years at enrolment and < 55 years at diagnosis<br>• females	• 124-item self-administered high school FFQ about adolescent diet (1960 – 1982)	• high sugar/SSB intake during adolescence positively associated with risk of adenoma<br>• per 5%-increase of calories from total fructose intake OR 1.17 (95% CI 1.05 – 1.31)<br>• per 1 SSB/day increase OR 1.11 (95% CI 1.02 – 1.20)<br>• sugar/SSB intake during adulthood not associated with adenoma risk	• higher intake of simple sugars (e.g., fructose)<br>• higher intake of SSB
Kim et al.<br>(2021)<br>USA	• EO-CRC cases: n = 111<br>• follow-up: 1 250 560 person-years<br>• 25-42 years at enrolment and < 50 years at diagnosis<br>• females	• validated, semi-quantitative FFQ every 4 years	• higher total vitamin D intake significantly associated with reduced EO-CRC risk (HR for ≥450 IU/day vs <300 IU/day 0.49; 95% CI, 0.26 - 0.93)<br>• HR per 400 IU/day increase 0.46 (95% CI 0.26 - 0.83)<br>• inverse association significant	• low vitamin D intake
Nguyen et al.<br>(2021)<br>USA	• EO-adenoma cases: n = 2911<br>• 25-42 years at enrolment and < 50 years at diagnosis<br>• females	• validated, semi-quantitative FFQ every 4 years	• higher sulfur microbial diet scores associated with increased risk for EO-adenomas (OR Q4 vs.Q1 1.31, 95% CI 1.10 - 1.56)	• higher intake of processed meats<br>• low intake of vegetables and legumes

		• high school FFQ (adolescent diet (1960 – 1982))<br>• composition of a sulfur microbial diet score	• similar trends for EO-adenoma based on diet consumed during adolescence	
Zheng et al. (2020) USA	• EO-adenoma cases: n = 1157<br>• 25-42 years at enrolment and < 50 years at diagnosis<br>• females	• validated, semi-quantitative FFQ every 4 years<br>• derivation of dietary patterns	• Western diet positively associated with risk of EO-adenoma<br>• prudent diet, DASH, AMED, and AHEI-2010 inversely associated with risk of EO-adenoma<br>• for high-risk adenomas, the highest vs. lowest quintile: Western OR 1.67, 95% CI 1.18 - 2.37; prudent OR 0.69, 95% CI 0.48 - 0.98; DASH OR 0.65, 95% CI 0.45 - 0.93; AMED OR 0.55, 95% CI 0.38 - 0.79; AHEI-2010 OR 0.71, 95% CI 0.51 - 1.01	• westernized dietary pattern
**Case-control studies**				
Deng et al. (2023) China	• EO-CRC cases: n = 811<br>• controls: n= 945<br>• < 50 years<br>• females + males	• validated, semi-quantitative FFQ	• highest vs. lowest sweet food and fried food intake both significantly associated with increased EO-CRC risk (OR sweet 2.70, 95% CI 1.89–3.86; OR fried 2.16, 95% CI 1.29–3.62)	• higher intake of sweet foods (e.g., dessert, SSB)<br>• higher intake of fried foods
Pan et al. (2023) China	• EO-CTC cases: n = 222<br>• controls: n = 87833<br>• < 50 years<br>• females + males	• FFQ interview	• EO-CRC associated with lower fish intake, monthly vs. weekly (HR 1.64; 95% CI 1.01 - 2.67)	• lower intake of fish
Puzzono et al. (2022) Italy	• EO-CRC cases: n = 47<br>• controls: n = 71<br>• 18 – 49 years<br>• females + males	• validated, semi-quantitative questionnaire	• fresh meat (p = 0.003), processed meat (p < 0.001) and dairy products (p = 0.013) significantly associated with EO-CRC	• higher intake of red meat<br>• higher intake of processed meats<br>• higher intake of dairy products
Archambault et al. (2021) Asia, Australia, Canada, Europe, USA	• EO-CRC cases: n = 3767<br>• controls: n = 4049<br>• < 50 years<br>• females + males	• FFQ	• EO-CRC associated with higher red meat intake (OR 1.10, 95% CI 1.04 - 1.16)<br>• lower total fiber intake linked more strongly to rectal (OR 1.30, 95% CI 1.14 - 1.48) than colon cancer (OR 1.14, 95% CI 1.02 - 1.27)	• low fiber intake<br>• low folate intake<br>• low calcium intake<br>• higher intake of red meat

			• lower folate consumption and EO-CRC risk OR 1.14, CI 1.04 – 1.24 for colon cancer; OR 1.24, CI 1.11 – 1.37 for rectal cancer<br>• low calcium intake and EO-CRC risk OR 1.15, CI 1.05 – 1.26 for colon; OR 1.24, CI 1.11 – 1.39 for rectum	
Chang et al.<br>(2021)<br>Canada	• EO-CRC cases: n = 175<br>• controls: n = 253<br>• 20-49 years<br>• females + males	• online questionnaire including consumption of various foods as a FFQ for the time period 2 years previously<br>• derivation of a Western-like dietary pattern score	• ≥ 7 vs. < 1 SSB drinks/per week OR 2.99; 95% CI 1.57 – 5.68) associated with an increased risk of EO-CRC<br>• westernized dietary pattern (quartile 4 vs. 1, OR 1.92; 95% CI 1.01 – 3.66) associated with increased risk of EO-CRC<br>• calcium supplement use (OR 0.53; 95% CI 0.31 – 0.92) associated with reduced risk of EO-CRC	• higher consumption of SSB<br>• westernized dietary pattern<br>• low calcium intake
Khan et al.<br>(2015)<br>Pakistan	• EO-CRC cases: n = 74<br>• controls: n = 148<br>• 18 – 65 years<br>• females and males	• structured questionnaire about dietary practices	• individuals with a high-fat diet had a 98% higher chance for EO-CRC compared to those who did not (OR 1.98, 95% CI 1.13 - 3.49)	• high-fat diet
Rosato et al.<br>(2012)<br>Italy, Switzerland	• EO-CRC cases: n = 329<br>• controls: n = 1361<br>• < 46 years<br>• females + males	• validated FFQ	• OR of EO-CRC were 1.56 for the highest tertile of processed meat, 0.40 for vegetables, 0.75 for fruit, 0.78 for fish, 0.52 for beta-carotene, 0.68 for vit. C, 0.38 for vit. E and 0.5 for folate	• higher intake of processed meat<br>• low intake of vegetables<br>• low intake of fruit<br>• low intake of fish<br>• low intake of beta-carotene<br>• low intake of vitamin C<br>• low intake of vitamin E<br>• low intake of folate
